# Reduction in EEG theta power as a potential marker for spatial disorientation during flight

**DOI:** 10.1038/s41598-025-85219-4

**Published:** 2025-01-11

**Authors:** Gil Geva, Nir Getter, Boris Blecher, Oded Ben-Ari, Barak Gordon, Idan Nakdimon, Oren Shriki

**Affiliations:** 1 Department of Cognitive and Brain Sciences, Ben-Gurion University of the Negev, 1 Ben-Gurion Blvd, Beer-Sheva, Israel; 2 The Israeli Air Force Aeromedical Center, Tel-Hashomer, Ramat Gan, Israel; 3 Israeli Defence Forces Medical Corps, Tel-Hashomer, Ramat-Gan, Israel; 4 Department of Military Medicine, Faculty of Medicine, The Hebrew University, Jerusalem, Israel

**Keywords:** Sensory processing, Neuroscience, Perception

## Abstract

During flight, spatial disorientation (SD) commonly occurs when a pilot’s perception conflicts with the aircraft’s actual motion, attitude, or position. A prevalent form of SD is the somatogyral illusion, which is elicited by constant speed rotation and causes a false perception of motion in the opposite direction when the rotation ceases. This research aimed to investigate changes in brain activity that occur when experiencing a somatogyral illusion by simulating conditions closely mimicking flight conditions to gain insight into how to better manage this illusion during flight. In the study, 23 volunteers were isolated from external stimuli to promote somatogyral illusion induction while seated in a Barany (rotating) chair. The study employed electroencephalogram (EEG) and eye-tracking glasses to monitor brain activity and eye movements, respectively. Participants reported their perceived motion direction using a joystick, allowing us to compare a reference condition to that of the illusion. Results indicate a significant decrease (34%) in theta power (4–7.5 Hz) over the left frontal region during the illusion, complemented by the occurrence of nystagmus in 72% of the trials. These findings align with previous studies linking SD and theta band changes, suggesting implications for EEG-based identification of SD in flight.

**Supplementary Information:**

The online version contains supplementary material available at 10.1038/s41598-025-85219-4.

## Introduction

Spatial disorientation (SD) is a persistent challenge in aviation that has contributed to numerous accidents, loss of lives, aircraft damage, and reduced mission effectiveness^1^. Despite significant technological advancements in the aviation field over the past century, the human body, with its evolutionary adaptation to a terrestrial 1G environment directed toward Earth’s center, has remained fundamentally unchanged. Accurate perception of orientation and movement in space is crucial for pilots to operate aircraft effectively and safely. However, pilots, like all humans, are physiologically ill-equipped to handle the dynamic and complex flight environment. Instances of pilots emerging from dark clouds or fog only to find themselves flying at an incorrect angle or even upside down are not uncommon. Despite decades of awareness within the aviation community, the rate of SD-related accidents has not significantly decreased. In fact, SD accounts for approximately one-third of all aviation mishaps^2^, with high mortality rates in both military and general aviation^3^.

During flight, the brain may receive conflicting information from multiple sensory systems—primarily visual and vestibular—which lead to SD. Of the various types of vestibular illusions, the *somatogyral* illusion is particularly significant^1,4–6^. This illusion creates a false sensation of rotational movement when none exists, or conversely, a lack of sensation when rotational movement is occurring^7,8^. Moreover, an *oculogyral* illusion, often a consequence of a somatogyral illusion, can result in uncontrollable lateral eye movements^1^ that further exacerbate a pilot’s ability to maintain spatial orientation.

**Fig. 1:**
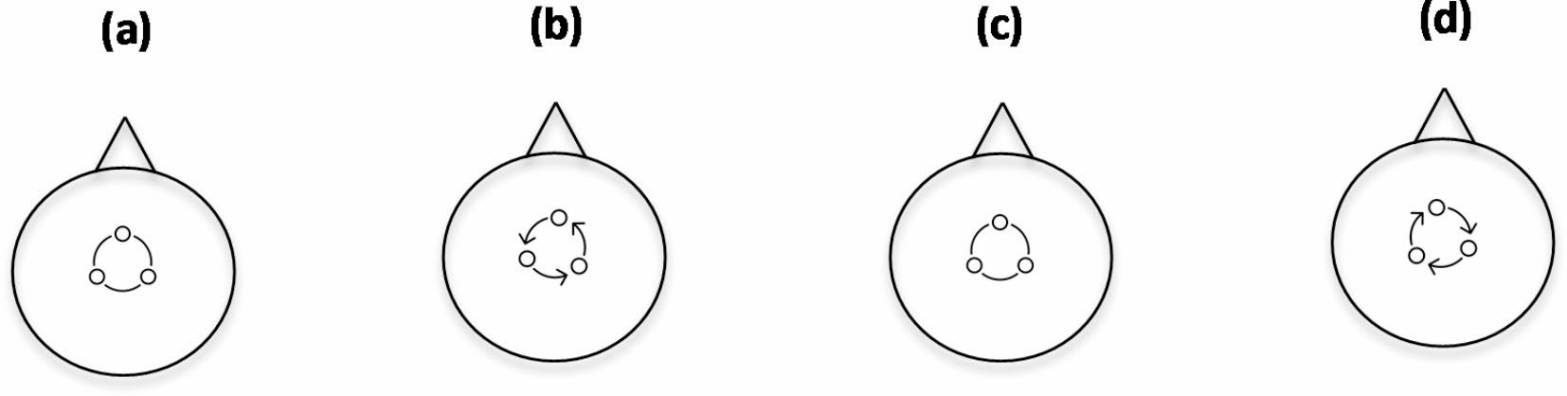
Mechanism of the somatogyral illusion. This schematic of a subject viewed from above depicts the phases of the illusion. (**a**) No turning: the subject reports no sensation of movement. (**b**) Initiation of turn: a sensation of turning arises as the fluid in the horizontal semicircular canal deflects the hair cells within the cupula. (**c**) During a constant rate of turning, the sensation ceases as the fluid velocity matches the canal wall. (**d**) Upon stopping the turn, a sensation of turning in the opposite direction occurs as the fluid in the ear canal continues moving, now deflecting the hairs in the opposite direction.

Figure 1 illustrates the dynamics of a somatogyral illusion. In this scenario, a subject whose vision is obscured and who initially feels no movement (a) experiences rapid counterclockwise yaw acceleration, eventually stabilizing at a constant rotational speed around a vertical axis (b). This movement activates the “yaw canals.” Within approximately 15 to 60 s, the sensation of counterclockwise motion fades, allowing the subject to feel stationary once more (c). However, when the rotation ceases, the subject experiences a sensation of turning in the opposite direction (d). This prolonged perception of self-rotation, occurring even though the subject remains physically stationary, extends beyond the cupula’s time constant in the yaw semicircular canals. This phenomenon is attributed to the velocity storage mechanism, as detailed in seminal studies by Cohen and colleagues^9,10^.

The detection of unrecognized SD through objective markers is crucial in order to reduce its associated risks. Real-time alerts about the occurrence of this phenomenon can empower pilots to take countermeasures, such as using flight instruments, engaging autopilot systems, or initiating a “go around” procedure^8^.

EEG-based metrics can identify changes in neural dynamics that correspond to SD and play a significant role in this endeavor. A particularly promising EEG metric is the power within the theta frequency band (4–7.5 Hz), which is closely associated with spatial orientation. Generated in the hippocampus^11^, theta rhythms are essential to spatial navigation and memory encoding. They are thought to provide a temporal framework for organizing movement sequences, memory encoding, and planning trajectories in spatial navigation^11,12^. Research has consistently linked hippocampal theta rhythms to memory formation^2,8^ and navigation^7,13,14^ in humans and animals. Furthermore, previous studies have demonstrated changes in the theta band during visually-induced unrecognized SD^15^.

Recent advancements in this field include the work of Chen et al., which highlights the role of theta oscillations in the medial prefrontal cortex (mPFC)–entorhinal cortex (EC) circuit in facilitating spatial computations for human navigation^16^. Similarly, Berger’s study reveals similarities between the brain signals produced during spatial navigation and episodic memory recall, with low-frequency theta rhythms appearing as individuals navigate between memories^17^.

Another means of objectively recognizing SD is by analyzing eye movements. One significant phenomenon—*nystagmus*, which refers to involuntary, rapid, and repetitive eye movements—is often indicative of a dysfunction in the vestibular system^18^. Nystagmus is particularly relevant to SD because it can be induced by conditions that disorient the sensory systems, such as the rapid or unusual movements often experienced during flight. The presence of nystagmus can serve as a physiological indicator of the body’s attempt to reconcile conflicting sensory information related to movement and orientation, making it a valuable objective marker for detecting SD^19,20^.

In our study, we aimed to identify objective markers of the somatogyral illusion in controlled settings through the use of EEG and eye-tracking technologies. A significant motivation for our research was the need to delve into the neurophysiological mechanisms of SD under dynamic conditions—as existing research has primarily concentrated on illusions which are static^15^.

Our experimental paradigm was designed to induce the somatogyral illusion. Twenty-three volunteers participated in the study, in which they were seated in a rotating (Barany) chair capable of precise control. The participants experienced rotations and sudden stops, during which their brain activity was monitored via a wireless EEG system, and their eye movements were captured using an eye-tracking device. To effectively induce the illusion, we isolated the subjects from external visual and auditory inputs. Participants reported their subjective experiences of the illusion—whether they felt a sensation of rotation or its absence—by moving a joystick in the perceived direction of motion or by returning it to the center to indicate that they felt no movement sensation. Our methodology involved conducting within-subject comparisons, contrasting brain and eye activity during a reference condition (before chair rotation) with that observed during the illusion (an induced sensation of motion following termination of the rotation).

The distinctiveness of our research stems from our focus on examining alterations in the EEG theta signal and eye movements as indicators of SD, specifically in the context of the somatogyral illusion. By analyzing these combined changes, we aimed to provide insights into the brain’s response to disorienting stimuli and the associated eye movements, offering a novel perspective on the detection and understanding of SD.

The integration of eye-tracking technology with an EEG data analysis presents a promising avenue for detecting SD, especially in terms of identifying somatogyral illusions. This approach has the potential not only to enhance our understanding of the neurophysiological responses to SD, but also to inform development of diagnostic tools and interventions. By identifying specific patterns of eye movement and EEG signals associated with SD, we can improve the safety and efficacy of pilot training programs, develop more effective countermeasures against SD, and ultimately contribute to reducing the incidence of aviation accidents attributed to SD. The findings from this study may also have broader implications for the study of vestibular disorders and their impact on spatial orientation and movement perception, paving the way for interdisciplinary research and applications beyond aviation safety.

## Methods

### Subjects

Twenty-three healthy male subjects (mean age 21 ± 1.7 years), all serving in the Israeli Defense Forces (IDF), participated in the study after giving written informed consent. All participants were right-handed, had normal (or corrected-to-normal) vision, and reported no history of neurological or psychiatric disorders. Prior to the test, all of the subjects were physiologically examined by an expert otolaryngologist to eliminate any undetected abnormalities. The study was approved by the Institutional Review Board of the IDF, and all experiments were performed in accordance with relevant guidelines and regulations.

### Equipment

**Fig. 2:**
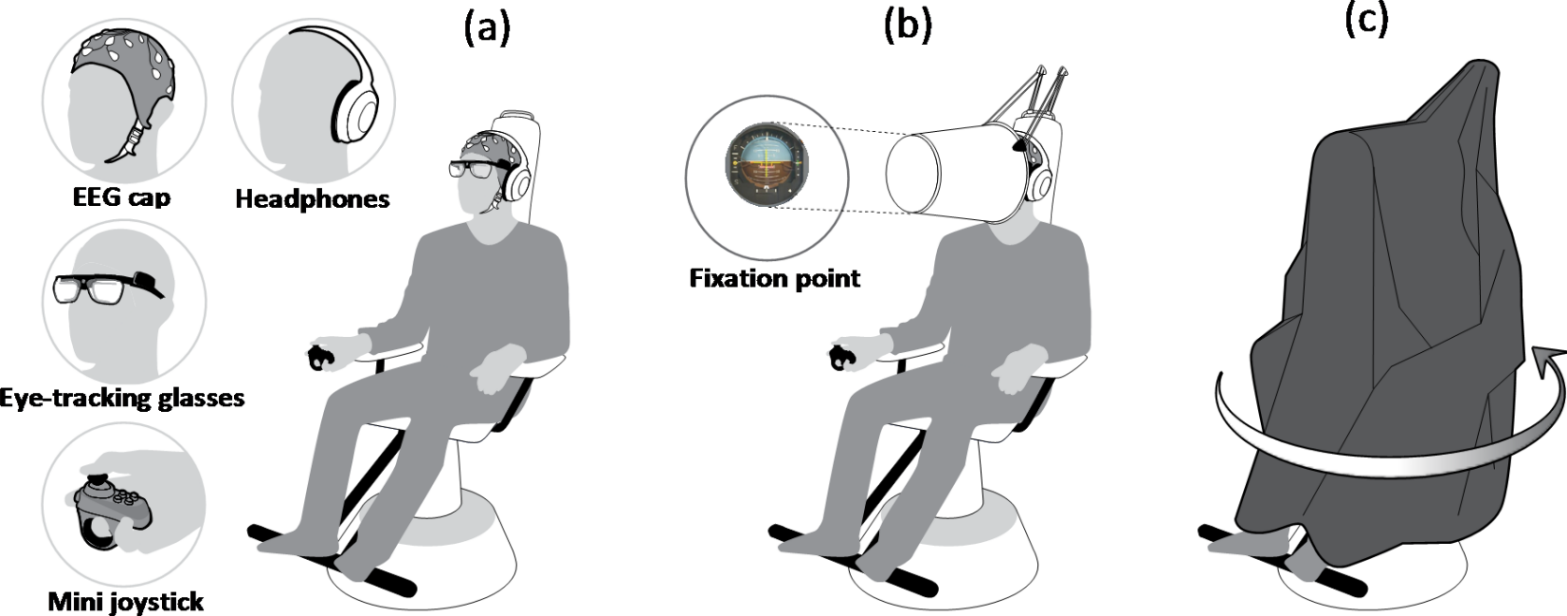
Experimental setup. Subjects were seated on a rotating Barany chair. (**a**) Recording apparatus mounted on the subject’s head and mini joystick held in the right hand to signal rotation direction when sensed. (**b**) Field of view was restricted, and subjects were visually obscured from the outside world, with their vision on a fixation point. (**c**) A double layer of dark lightweight fabric insulated the subjects from any other visual cues.

We recorded EEG and eye-tracking data from the subjects during exposure to a vestibular illusion. The EEG data were recorded using a g.Nautilus wireless biosignal acquisition 10–20 system with 32 active dry electrodes (gTec, Austria). Eye-tracking data were collected using a Tobii Wearable Wireless “Pro Glasses 2” system with a sampling rate of 100 Hz. Eye movement monitoring was used to identify nystagmus (involuntary rapid eye movement), a marker of SD^3,19–21^. Subjects were first seated on a rotating Barany chair (Fig. 2a). After setting up the EEG cap on each participant, we adjusted the eye-tracking glasses and sound-canceling headsets, which emitted white background noise (to eliminate the sound of the chair motor). In their hand, subjects held a small wireless joystick in order to convey their subjective feeling about the direction of the motion they perceived (right, left, or no motion). Subsequently, they were visually obscured from their surroundings by a bucket-shaped device. They were instructed to maintain their fixation point at the center of an illuminated image of an attitude indicator cross, which was displayed at eye level at a distance of 40 cm (Fig. 2b). Following this, subjects were surrounded with a double layer of dark lightweight fabric, which covered and insulated them from any other visual cues, such as outside lights (Fig. 2c). Motion-sensitive cameras were used to monitor the rotation of the Barany chair and to identify the exact time points when it began and ended rotating. The data streams coming from all sensors were synchronized to the same timeline using MATLAB Simulink^®^ (see Supplementary Information S1 for a detailed layout of the Simulink panel configuration).

### Procedure

**Fig. 3:**
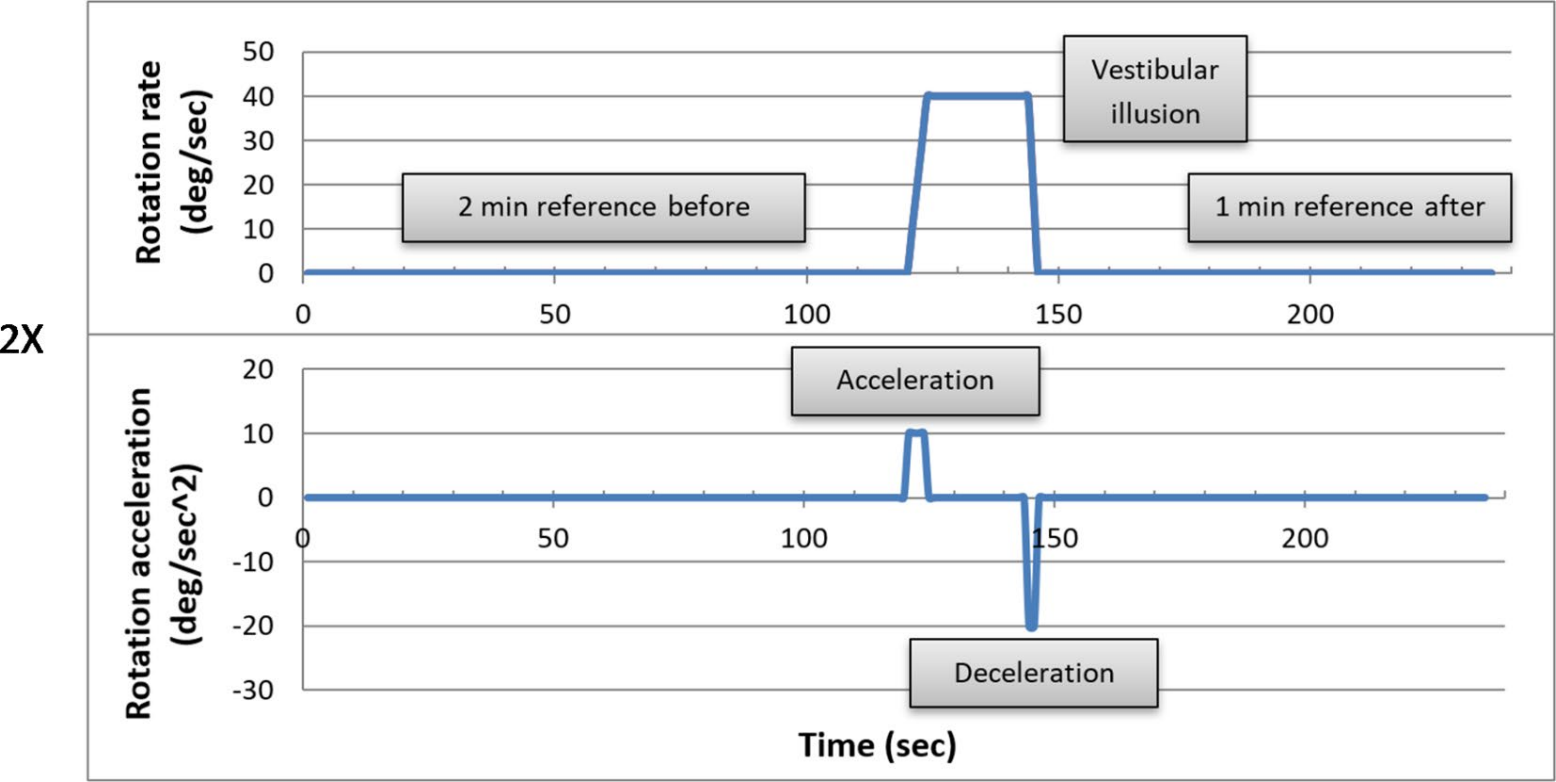
Experimental protocol. Two sets of consecutive repetitions were performed for each subject. Each included a 2-min stationary reference state, followed by a slow angular acceleration up to a steady rate. A somatogyral illusion was present when the rotating chair was abruptly stopped, and the subject reported a sense of movement by signaling the rotation direction with the wireless joystick. A second stationary reference phase data recording concluded the protocol.

The sequence states of the Barany chair (Fig. 3) included a stationary state, a rotation state, and a second stationary state. The first stationary state lasted 2 min and represented a reference time window with no objective motion and no subjective feeling of motion. The rotational state comprised three distinct phases: acceleration, sustained rotation at a constant angular velocity, and deceleration. It was initiated with a counterclockwise (left-handed) circular acceleration, characterized by a rate of 10 degrees per second squared (10 deg/sec^2^). This acceleration phase persisted for a duration of 4 s, resulting in the attainment of an angular velocity of 40 degrees per second (40 deg/sec). Subsequently, the system entered a stable, continuous rotation phase, maintaining a constant angular velocity of 40 degrees per second (40 deg/sec) for a duration of 20 s. Finally, the deceleration phase ensued, promptly bringing the chair to a complete halt within 2 s, facilitated by a deceleration rate of 20 degrees per second squared (20 deg/sec^2^). The second stationary state had a variable subject-dependent duration. Following the stop of the rotation, subjects felt illusory motion in the opposite direction (typically, for about 20 s). Once they reported no motion using the joystick, we continued recording for another 60 s, which represented a second reference time window. All of the above numeric values were target values, and the range of accuracy within the experiment was up to ± 5%. All subjects were exposed to two consecutive repetitions of the above experimental sequence (Run#1 & Run#2). EEG data were collected throughout all of the experimental states, as was the eye-tracking data which was tested and calibrated prior to each run.

### Nystagmus

Nystagmus was identified by visually inspecting the data from all runs, based on the following criteria: the direction of the sawtooth wave was based on the direction of rotation— the expected pattern involved a rapid shift to the right followed by a gradual return to the left. The period was around 1 s with an amplitude of more than four degrees (> 4°). The phenomenon duration extended for more than 5 s, with a fadeout phase.

### Data analysis

#### Pre-processing

We analyzed the data using MATLAB^®^ software with an in-house code as follows: Data were first high-pass filtered (cut-off 1 Hz), then a customized adaptive filter was applied to suppress line noise. This was followed by Artifact Subspace Reconstruction^22^, re-referencing to the EEG mean, and low-pass filtering (cut-off 40 Hz). Next Infomax ICA was carried out^23^. The resulting independent components (ICs) were evaluated automatically for artifacts by combining spatial, spectral, and temporal analyses. ICs identified as containing ocular, muscular, or cardiac artifacts were removed from the data. To reject the possibility of a repetition effect, we applied a t-test to the indicated SD time for each subject between the first and second run.

#### Spectral analysis

To make the analysis more robust, we chose to discard the transient 2-sec period that it takes the chair to reach a full halt. Thus, our analyses considered EEG data that were collected while the chair was at rest. To analyze the implications of SD on brain activity, we sampled a time window of 15 s from the SD period and a comparison time window of 15 s from the baseline period. Then, for each electrode, we computed the power spectral density (PSD) in each sample by means of a *pwelch* algorithm (window size 4 s, overlap 2 s). These PSDs were partitioned to power bands according to the common power-band definitions (Delta: 0.2–4 Hz, Theta: 4–7.5 Hz, Alpha: 7.5–12 Hz, Beta: 15–30 Hz, Gamma: 30–40 Hz).

#### Statistical analysis and cluster permutation test for multiple comparisons

Within each EEG frequency band, paired t-tests were conducted to compare baseline and SD conditions for each electrode separately (e.g., F3 theta during baseline vs. F3 theta during SD). To account for multiple comparisons across electrodes, a cluster-based permutation approach was performed using 10,000 permutations^24^. The analysis used a one-tailed test, focusing on the upper tail of the t-distribution. For the 3D cluster analysis, channel adjacency information was derived from the 32-channel EEG montage, creating a matrix that represented the spatial relationships among electrodes. Channel clusters were then formed by grouping adjacent electrodes that exceeded a critical t-value, corresponding to an alpha level of 0.01, calculated from the t-distribution’s percent point function (df = 24). The cluster-level statistic was computed as the sum of t-values within each cluster. Significance was determined by comparing the observed cluster-level statistics against the distribution of maximum cluster-level statistics obtained from the permuted data. The analysis was conducted using the permutation_cluster_test function from the MNE-Python library^25^.

#### Time-frequency analysis

To further analyze brain activity changes over time during SD, we chose electrodes for which the difference between the baseline and SD was significant (*p* < 0.05) for specific power bands. For the data from these electrodes, we computed a time-frequency analysis (TFA) matrix by means of wavelets. This analysis is sensitive to small deflections in the time-frequency domain. The SD matrix was then divided by the TFA from the baseline to obtain a matrix representing the change proportion between the baseline and SD, in which time zero represents the point in time where the SD was indicated by the subject and the chair was in the stop position.

## Results

**Fig. 4:**
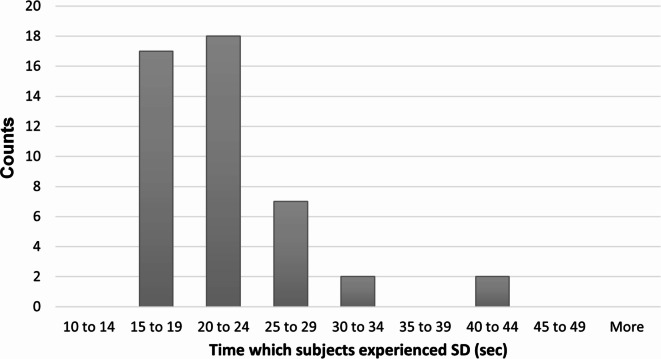
Subjective duration of perceived motion (sec). The histogram depicts the distribution across all trials of the duration of illusory perceived motion. The duration of the experiment was measured from the moment the rotating chair came to a full stop. The subject was instructed to report any subjective motion sensations experienced during this period. Altogether, 46 runs were documented.

EEG and eye-tracking data were collected from all of the subjects during the induction of the somatogyral illusion. Each participant experienced two consecutive repetitions of the experimental procedure (Fig. 3). The participants reported their sensation by moving the joystick to the side to which they felt like they were moving or holding it in a middle position whenever they sensed that they were steady. An analysis of their subjective indications about the chair’s rotation and direction demonstrated that all of the subjects experienced a somatogyral illusion for more than 15 s. Figure 4 depicts a histogram of the duration of perceived rotation following the abrupt stop of the chair rotation, as reported by the subjects throughout all 46 runs.

**Fig. 5:**
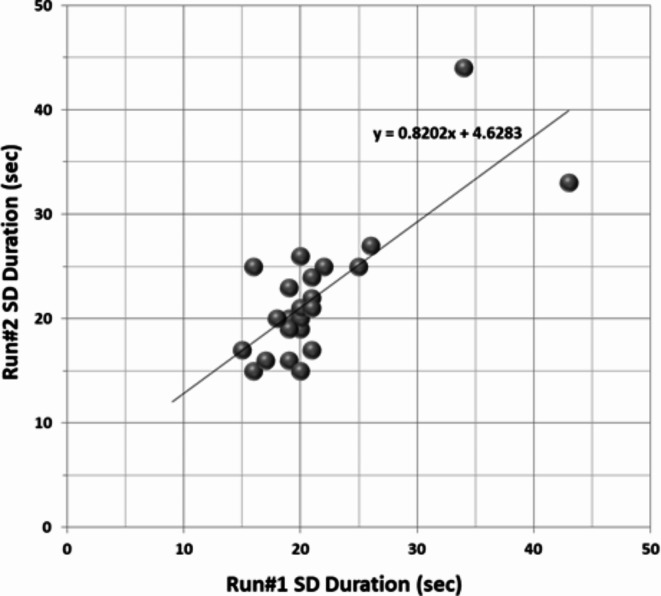
SD duration variability within subjects. Each dot represents a single subject’s time comparison for the duration of the perceived motion effect in Run#2 versus Run#1 (with time values rounded down to the nearest integer in seconds).

We tested for differences between the first trial (Run#1) and the second (Run#2), as subjects gained experience with the chair motions. No significant difference was found in the durations of perceived motion between the two runs (*p* = 0.88), indicating that there was no repetition effect. Figure 5 depicts a scatter plot of the durations of perceived motion in the two runs, showing small within-subjects variability.

As can be seen from Figs. 4 and 5, in 42 out of 46 trials, the perceived duration fell between 15 and 27 s. Two subjects reported significantly longer duration times compared to the average. Because this was consistent across both repetitions, we did not consider these results as outliers, but rather as a reflection of individual vestibular system sensitivity.

During the study, four different relative states were encountered in terms of the chair’s state and subjects’ reports (Table 1). The chair was either steady or in motion when each report was noted and recorded. We focused on EEG differences while subjects were at rest (stationary reference—mode 1) compared to the time frame when they reported false movement (somatogyral illusion—mode 3), as highlighted (bold) in Table 1.

**Table 1:** Four different states during the experiment.

Subject report/Chair state	Chair steady	Chair moving
Subject reported no movement	**1 – Stationary reference**	2 – Illusion
Subject reported movement	**3 – Illusion**	4 – Motion reference

Analyses compared state 1 (stationary reference) to state 3 (somatogyral illusion). These states are highlighted in bold.

The data from Run#1 and Run#2 were analyzed independently. The analysis focused on the 2-min period prior to the chair’s initiation of movement (stationary reference) and the period commencing 2 s after the participants reported experiencing a sensation of movement. The participants indicated their perception of movement as soon as the deceleration began. To enhance the robustness of our analysis, we excluded the transient 2-sec interval required for the chair to come to a complete stop. Therefore, our analysis was confined to EEG data collected only while the chair was stationary. A time frame of 15 s was sampled from each state for further analysis.

**Fig. 6:**
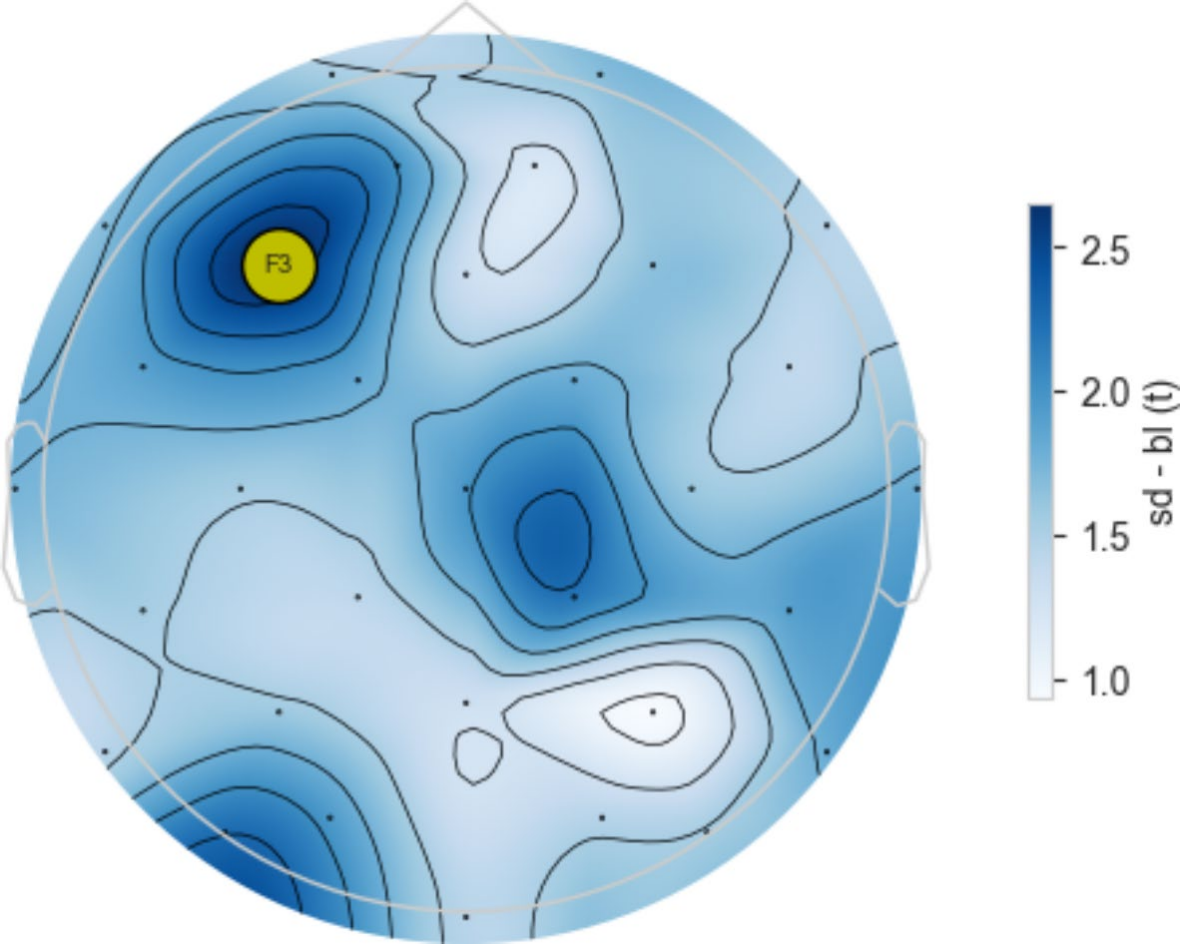
Topographic plot of t-values and cluster permutation analysis for the theta band. Blue shades represent t-values comparing the Spatial Disorientation (SD) condition to the baseline, as indicated by the color bar on the right. Each small dot marks a channel location in the 10–20 EEG system. The F3 channel is highlighted in orange, indicating it met the significance threshold of *p* < 0.05 according to the cluster permutation analysis.

For each combination of channel and frequency band, we tested the change in relative power between the two conditions and evaluated statistical significance. To account for multiple comparisons across electrodes, we applied a cluster permutation test (see Methods). Statistically significant results (*p* < 0.05) were observed only in the theta band. Within this band, we identified one significant cluster, which comprised a single electrode, F3. This is depicted in Fig. 6 which presents a topographic plot of the t-values for the theta band in Run #1. No significant clusters were identified in all other frequency bands (delta, alpha, beta, gamma).

**Fig. 7:**
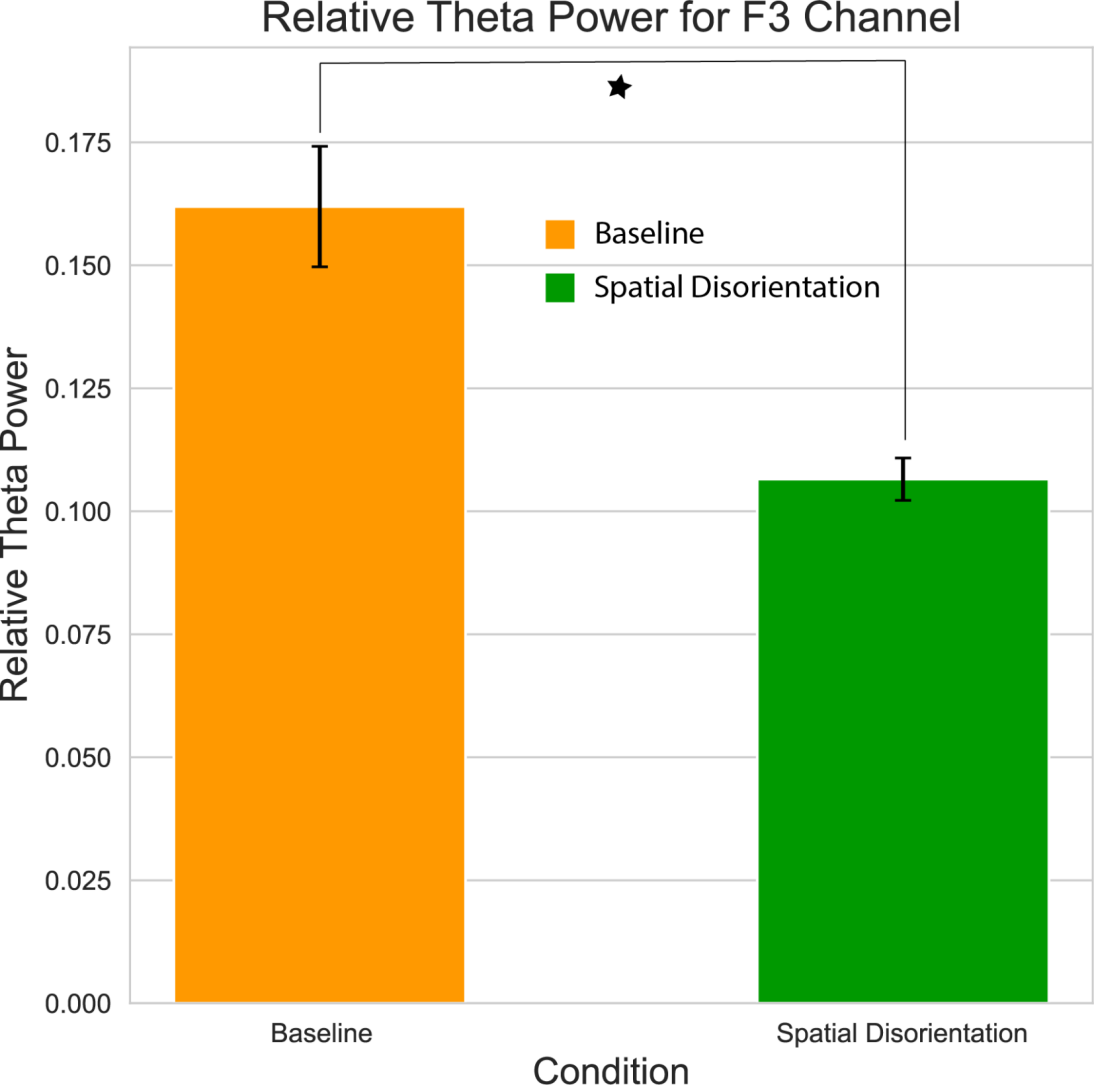
Theta band relative power difference recorded at electrode F3 during Run #1. The left bar represents the baseline condition, while the right bar represents the Spatial Disorientation (somatogyral illusion) condition. The mean relative theta power decreased by 34% during the illusion. A statistically significant difference (*p* < 0.05) was identified using the cluster permutation test. Error bars represent the Mean Squared Error (MSE).

Following the clustering analysis, we focused our analysis on relative theta power (RTP) in channel F3. We analyzed the effect of the somatogyral illusion on the RTP in comparison to the baseline state. As depicted in Fig. 7, there was a global decrease by ~ 34% in the theta power (mean baseline = 0.16RTP, mean SD = 0.10RTP). The same statistical analysis was conducted on the data from Run #2, but no significant effects were found.

**Fig. 8:**
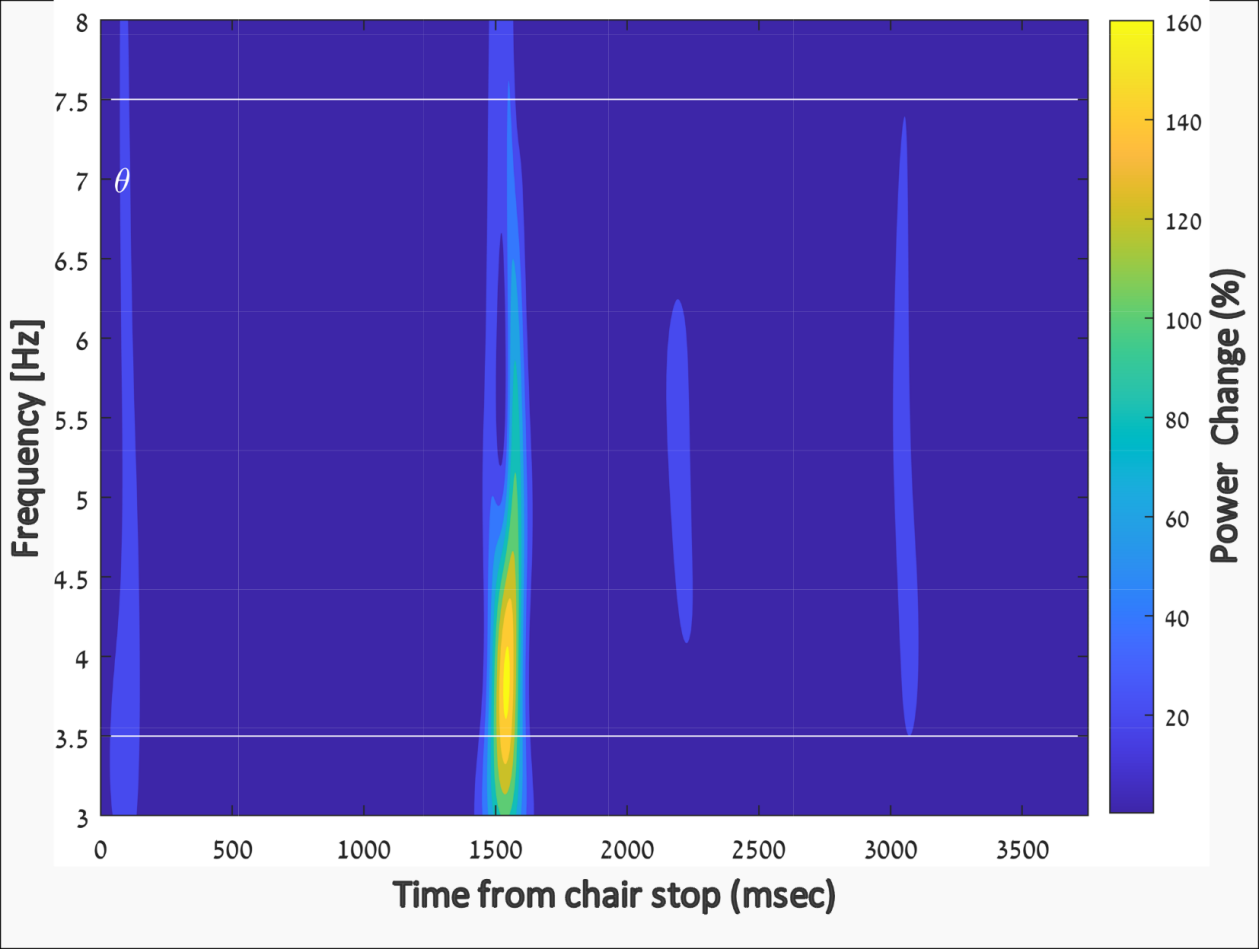
Relative reduction in power over time during the somatogyral illusion. A significant reduction in low theta (θ) band power is observed in electrode F3 at 1.5 s after the chair reaches the stop position (onset of the illusion). The color coding represents the relative reduction in power compared to baseline. The white horizontal lines represent the frequency range of the theta band.

To evaluate the temporal dynamics of the power change during the illusion, we applied a time-frequency analysis to the power changes in electrode F3 relative to the baseline condition (Fig. 8; grand average across all subjects in Run#1). As can be seen, a significant reduction in the lower theta band power was present around 1.5 s after the chair came to a halt (time 0).

**Fig. 9:**
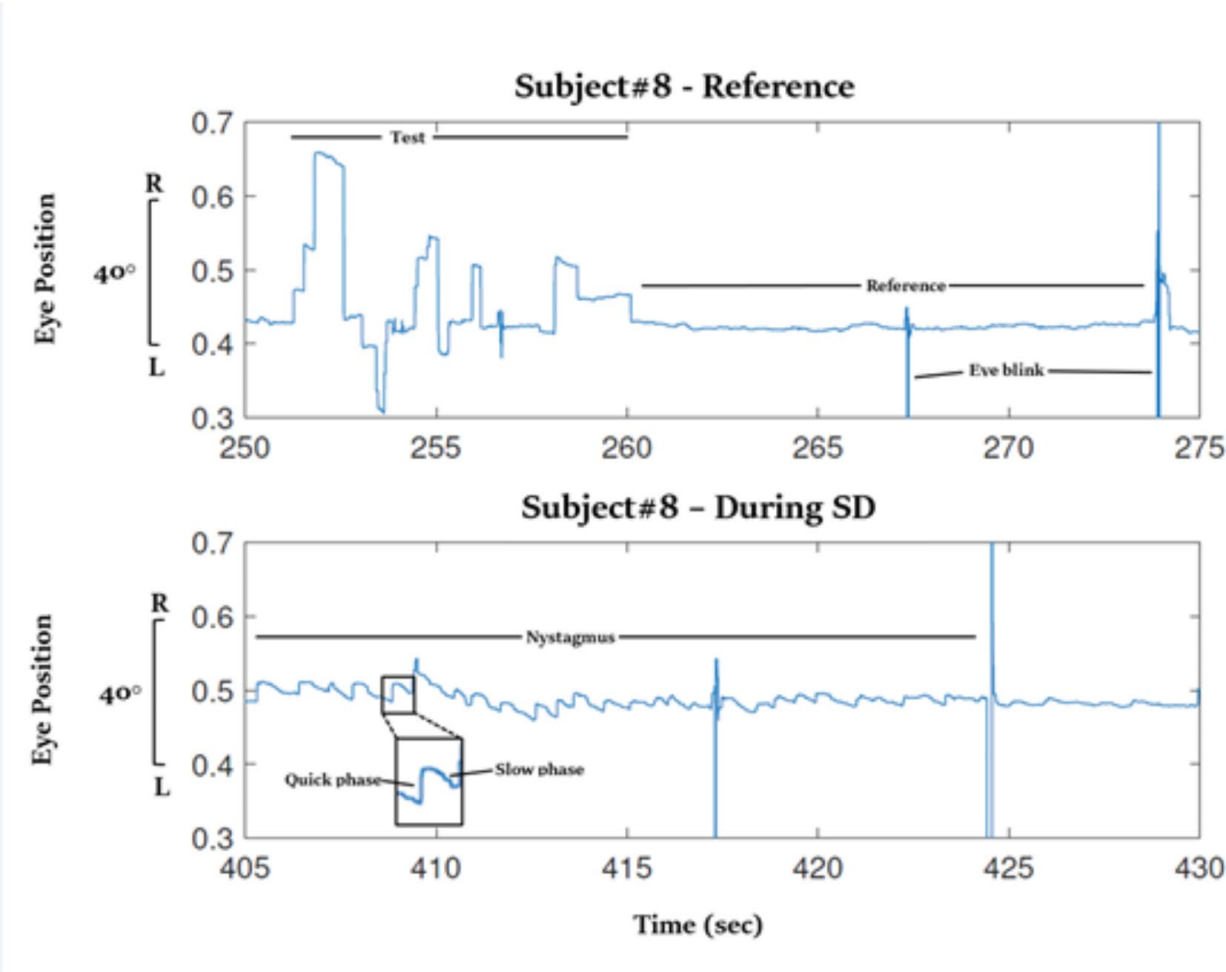
Nystagmus during SD. The figure depicts the horizontal eye position for Subject#8. Upper panel – during the calibration state, while the chair was steady, his eye position diverted left and right, and from ~ 260 s and on, it was stable on a fixation point, with two blinks followed at 267.5 and 274 s. Lower panel – 2 s after the chair was stopped from rotating in a counterclockwise direction, a vestibular nystagmus can be traced from the 405-sec point. In the beginning, his eyes moved slowly at the same speed as the chair (slow phase), and occasionally, made rapid resetting movements (quick phase). The speed of the slow phase gradually decreased until it faded away toward the end of the somatogyral illusion. The units for eye position are defined such that 0.5 is the center of the visual field, and a change of 0.1 corresponds to 20 degrees from the center position.

The tested somatogyral illusion, as reported by the subjects, was accompanied at most trials (72%) by unilateral involuntary repetitive eye movements (nystagmus). When this occurred, a two-phase eye movement was identified after the abrupt stop of the chair was activated—a slow phase in the same direction that the chair had rotated and a quick phase in the opposite direction. Figure 9 demonstrates the phenomenon as recorded from subject#8 when he experienced a somatogyral illusion after the chair stopped moving in a counterclockwise rotation. As the direction of nystagmus is defined in clinical practice by the direction of its quick phase, this led to a leftward slow phase and a right-beating nystagmus. Eventually, as the sense of motion vanished, the nystagmus decayed, and the eye movement returned to its baseline rate, as performed throughout the reference session.

## Discussion

In this study, we investigated the utility of EEG in detecting unidentified spatial disorientation, specifically, the somatogyral illusion. The main goal of the study was to record data while the subject was in motion, mirroring as closely as possible the actual conditions that would be experienced in flight. Our findings reveal that statistically significant changes in EEG measures occurred as a consequence of the somatogyral illusion. Specifically, theta power in electrode F3 diminished, correlating with the illusion. Regarding eye movement, nystagmus was observed in the majority (72%) of all somatogyral illusion runs. This study also highlights the effectiveness of using lightweight, wearable EEG and eye-tracking systems for data collection in SD conditions.

After the chair was abruptly stopped, all of the subjects, with no exceptions, reported a strong sensation of rotation for a significant time (more than 15 s in all 46 trials); however, conclusive and statistically significant findings were established only in the first run out of the two. This is most likely due to participants setting up prior expectations during the second exposure. Moreover, we did not see the nystagmus effect in all trials, but only in 72% of all incidences where the somatogyral illusion was present. The proportion of trials where no nystagmus occurred was similar across the two runs. This suggests that the presence of this phenomenon was less affected by subjects’ expectations, and was more consistent than the reduction in theta power.

In our experiment, the somatogyral illusion effect was triggered by an abrupt stop (in less than 2 s) of the Barany chair. Subjects described the illusion as a sudden rise in rotation sensing that faded as time passed. The impact of the first second after the chair came to a halt was more substantial and meaningful than at any other time, and after 15 s or more, the illusion dissolved, and the subject signaled a steady position, balancing his sensation with the chair position. This behavioral analysis is corroborated by the theta power dynamics revealed through the time-frequency analysis.

In past years, there were very few studies that tested EEG brain activity in conjunction with exposure to vestibular illusions. Interestingly, even so, the existing studies suggest an association between spatial disorientation and changes in the theta band that are consistent with the present findings. In 1999, Tokumaru et al. reported a lack of statistical evidence in brain wave recordings that accompanied vestibular illusion phenomena^4^. In contrast, Stephens et al. (2003) reported a significant effect on theta waves during the formation of a Coriolis illusion^26^. Moreover, a study by Horng et al. (2009) showed a clear increase in peak alpha frequency during exposure to this illusion^21^. Specifically, a value of 11.94 ± 1.20 Hz before exposure increased to a value of 13.15 ± 0.84 Hz during exposure to the phenomenon. In another study, Li et al. (2015) reported that during exposure to a visually-induced oculogyric illusion, a decrease in certain EEG-based measures of brain connectivity in the theta frequency band occurred^15^. These finding are consistent with the assertion of Kahana et al. (1999) and Ekstrom et al. (2005), stating that human theta oscillations exhibit task dependence during virtual maze navigation^27,28^. Furthermore, multiple studies have shown that the theta band is a reliable marker for visually-induced motion sickness^29–32^. For example, Park et al. found that different subject groups (those who experienced motion sickness and those who did not) were significantly different with respect to the theta band at the frontal and parietal lobes^30^.

In terms of the neural circuitry involved in the observed effect, a prime candidate is the hippocampal–entorhinal system, which plays a vital role in spatial navigation and orientation. Studies in both animals and humans have demonstrated that theta oscillations in the hippocampus are closely associated with spatial tasks and movement, and are involved in spatial memory encoding and retrieval^12,33–36^. Therefore, it is plausible that changes in theta power during the somatogyral illusion reflect alterations in spatial perception and orientation. The false perception of motion induced by the illusion may disrupt the normal functioning of the hippocampal–entorhinal system and result in changes in theta oscillations. Although EEG is not directly sensitive to sub-cortical areas, it may still reflect relevant changes through connections between sub-cortical and cortical areas.

Because nystagmus is occasionally associated with SD, but is not present in all cases, our experimental result of only 72% out of all test runs is consistent with previous data. There were no obvious markers that could predict who, among of the subjects, would be affected by the phenomenon and who would not. The only possibility we can consider is that the chance of experiencing nystagmus appears to be higher when the surrounding light is dimmed and is highest in full darkness. When a subject’s head rotation stops abruptly, the endolymph fluid contained in the labyrinth of the inner ear continues to move in the same direction in which the head had formerly rotated. With a leftward rotation, this will inhibit the left horizontal canal and excite the canal on the right side, resulting in a sensation of rightward rotation and a corresponding right-beating nystagmus. However, this phenomenon occurs primarily in darkness. In the presence of light, optokinetic reflexes sustain nystagmus even as vestibular input wanes, provided the head continues to rotate. Correspondingly, these same reflexes also suppress post-rotatory nystagmus when there is light^37^. Therefore, to minimize the effect of these reflexes, during our experiment, we maintained conditions as close to dark as possible, while still allowing the fixation point to be identified by the subject. As we consider how the conditions in our experiment would translate into a real-life cockpit, it is more likely that pilot would experience a somatogyral illusion when there is some degree of light in the cockpit instruments, while the outside world may be totally dark or very dim.

In conclusion, we believe that it is essential to understand spatial orientation to comprehend how the human brain interprets the environment of flight in order to provide control and to prevent a loss in orientation that can lead to an accident. Moreover, alerting the pilot in real-time when spatial disorientation occurs can help him or her to take preventive actions such as engaging autopilot systems, or a pull-up or “go around” procedure. Our future goal is to extend these experiments into other types of SD illusions and to collect data from more subjects using an SD simulator. It would also be informative to investigate the interaction between SD and the cognitive workload, which is also associated with changes in theta power^38,39^. Thus, it is likely that unrecognized SD may lead to a high load on cognitive resources, and negatively affect a pilot’s performance.

Wearable EEG systems, already widespread in many applications, could easily be embedded in pilot helmets. Our study suggests that such a system could be used to detect SD during a flight and to generate appropriate alerts. Furthermore, eye-tracking devices are already mounted in many modern aircraft and are known as a precise and reliable technology that can identify the gaze point of the pilot, enabling a target to be locked onto with high precision. Integrating EEG with eye-tracking could further improve the reliability of such a system. Importantly, the relevant computations should be performed in real-time on a time scale of seconds, in contrast to the offline analysis presented here. However, modern technology allows these computations to be performed on short operationally relevant time scales.

A key point for this endeavor to succeed is understanding that every individual has their own pattern and regularity; thus, a baseline stamp recording should be performed for each pilot separately at the beginning of the monitoring phase, as well as throughout a mission in order to exclude abnormalities and to prevent false or nuisance alerts. In this way, our research uncovers only a glimpse of the potential for prolonged and active recordings that can enhance a system’s detection accuracy over time and recalibrate the setup whenever a new mission is begun.

While our study provides valuable insights into the understanding of SD, it does have several limitations. Among these, the use of subjective joystick reports from participants to gauge movement sensations may have been influenced by the velocity storage mechanism. This mechanism, known for its capacity to retain rotation information^10^, may have biased the reports of perceived motion. In contrast, the nystagmus response, elicited by motion stimuli, serves as a more objective measure within the framework of the study. In addition, research has illuminated gender-related differences in processing vestibular cues, with males and females demonstrating distinct responses^40^. This gender-based variance implies that our findings derived from a study group consisting exclusively of male participants may not be universally applicable across genders. Thus, future experiments should involve a mixed gender population. Another limitation of this study is that our research focused solely on one type of SD illusion—the somatogyral illusion. Future investigations should aim to explore a broader range of SD illusions to capture the complexity of this phenomenon more fully. Lastly, conducting the study in a laboratory setting, as opposed to during actual in-flight conditions, potentially oversimplifies the environmental factors that influence SD. Enhancing detection accuracy by incorporating a wider array of markers from EEG data, and additional sensors, along with the application of machine-learning tools represents a critical area for future research. Addressing these limitations is crucial to gaining more insight into SD and developing more effective diagnostic and intervention strategies.

## Electronic supplementary material

Below is the link to the electronic supplementary material.

Supplementary Material 1

## Data Availability

The data in this study are available from the corresponding author upon reasonable request.

## References

[1] Wickens, C. D. et al. Rotation Rate and Duration effects on the Somatogyral Illusion. *Aviat. Space Environ. Med.* **77**, 1244–1251 (2006). 17183920

[2] Gibb, R., Ercoline, B. & Scharff, L. Spatial disorientation: decades of pilot fatalities. *Aviat. Space Environ. Med.* **82**, 717–724 (2011). 21748911 10.3357/asem.3048.2011

[3] Cheung, B. & Hofer, K. Eye tracking, point of gaze, and performance degradation during disorientation. *Aviat. Space Environ. Med.* **74**, 11–20 (2003). 12546294

[4] Tokumaru, O., Kaida, K., Ashida, H., Yoneda, I. & Tatsuno, J. EEG topographical analysis of spatial disorientation. *Aviat. Space Environ. Med.* **70**, 256–263 (1999). 10102738

[5] Lewkowicz, R. & Biernacki, M. P. A survey of spatial disorientation incidence in Polish military pilots. *Int. J. Occup. Med. Environ. Health*. **33**, 791–810 (2020). 33029026 10.13075/ijomeh.1896.01621

[6] Pennings, H. J. M., Oprins, E. A. P. B., Wittenberg, H., Houben, M. M. J. & Groen, E. L. Spatial Disorientation Survey among Military pilots. *Aerosp. Med. Hum. Perform.* **91**, 4–10 (2020). 31852567 10.3357/AMHP.5446.2020

[7] Stott, J. R. R. Orientation and disorientation in aviation. *Extreme Physiol. Med.* **2**, 2 (2013). 10.1186/2046-7648-2-2 PMC3710190 23849216

[8] Heinle, M. T. E. & Ercoline, M. W. R. Spatial Disorientation: Causes, Consequences and Countermeasures for the USAF.

[9] Cohen, B., Matsuo, V. & Raphan, T. Quantitative analysis of the velocity characteristics of optokinetic nystagmus and optokinetic after-nystagmus. *J. Physiol.* **270**, 321–344 (1977). 409838 10.1113/jphysiol.1977.sp011955 PMC1353516

[10] Raphan, T., Matsuo, V. & Cohen, B. Velocity storage in the vestibulo-ocular reflex arc (VOR). *Exp. Brain Res.* **35**, 229–248 (1979). 108122 10.1007/BF00236613

[11] Nuñez, A. & Buño, W. The Theta Rhythm of the Hippocampus: from neuronal and Circuit mechanisms to Behavior. *Front. Cell. Neurosci.* **15**, 649262 (2021). 33746716 10.3389/fncel.2021.649262 PMC7970048

[12] Hasselmo, M. E. & Stern, C. E. Theta rhythm and the encoding and retrieval of space and time. *NeuroImage 85 Pt*. **2**, 656–666 (2014). 10.1016/j.neuroimage.2013.06.022 PMC3918488 23774394

[13] Lachaux, J. P., Rudrauf, D. & Kahane, P. Intracranial EEG and human brain mapping. *J. Physiol. Paris*. **97**, 613–628 (2003). 15242670 10.1016/j.jphysparis.2004.01.018

[14] Tesche, C. D. & Karhu, J. Theta oscillations index human hippocampal activation during a working memory task. *Proc. Natl. Acad. Sci. U. S. A.* **97**, 919–924 (2000). 10.1073/pnas.97.2.919 PMC15431 10639180

[15] Li, Y. et al. EEG functional network properties related to visually induced unrecognized spatial disorientation. *Biomed. Mater. Eng.* **26** (Suppl 1), S1115–1124 (2015). 26405869 10.3233/BME-151408

[16] Dong et al. Certain brain rhythms coordinate cognitive map in human spatial navigation. *Sci. Adv.* **7**, (2021).

[17] Berger Memory recall and spatial navigation elicit similar electrical activity in brain. *Penn Neurosci.* (2019).

[18] Davis, J. R., Johnson, R., Stepanek, J. & Fogarty, J. A. *Fundamentals Aerosp. Med.* (2011).

[19] Young, L. R. Optimal estimator models for spatial orientation and vestibular nystagmus. *Exp. Brain Res.* **210**, 465–476 (2011). 21416377 10.1007/s00221-011-2595-1

[20] Guerraz, M. et al. Visual vertigo: symptom assessment, spatial orientation and postural control. *Brain* **124**, 1646–1656 (2001). 11459755 10.1093/brain/124.8.1646

[21] Horng, C. T. et al. Changes in visual function during the Coriolis illusion. *Aviat. Space Environ. Med.* **80**, 360–363 (2009). 19378905 10.3357/asem.2173.2009

[22] Buzsáki, G. & Moser, E. I. Memory, navigation and theta rhythm in the hippocampal-entorhinal system. *Nat. Neurosci.* **16**, 130–138 (2013). 23354386 10.1038/nn.3304 PMC4079500

[23] Bigdely-Shamlo, N., Mullen, T., Kothe, C., Su, K. M. & Robbins, K. A. The PREP pipeline: standardized preprocessing for large-scale EEG analysis. *Front. Neuroinformatics*. **9**, 16 (2015). 10.3389/fninf.2015.00016 PMC4471356 26150785

[24] Maris, E. & Oostenveld, R. Nonparametric statistical testing of EEG- and MEG-data. *J. Neurosci. Methods*. **164**, 177–190 (2007). 17517438 10.1016/j.jneumeth.2007.03.024

[25] Gramfort, A. MEG and EEG data analysis with MNE-Python. *Front. Neurosci.* **7**, (2013). 10.3389/fnins.2013.00267 PMC3872725 24431986

[26] Stephens, M. S. *Electroneurophysiologic Diagnosis of Aircraft Pilot Spatial Disorientation* (Wright State University, 2003).

[27] Kahana, M. J., Sekuler, R., Caplan, J. B., Kirschen, M. & Madsen, J. R. Human theta oscillations exhibit task dependence during virtual maze navigation. *Nature* **399**, 781–784 (1999). 10391243 10.1038/21645

[28] Ekstrom, A. D. et al. Human hippocampal theta activity during virtual navigation. *Hippocampus* **15**, 881–889 (2005). 16114040 10.1002/hipo.20109

[29] Liu, R., Xu, M., Zhang, Y., Peli, E. & Hwang, A. D. A pilot study on EEG-Based evaluation of visually Induced Motion Sickness. *J. Imaging Sci. Technol.* **64** (1-), 20501 (2020). 10.2352/J.ImagingSci.Technol.2020.64.2.020501 PMC8075303 33907364

[30] Park, J. R. et al. Long-term study of simulator sickness: differences in EEG response due to individual sensitivity. *Int. J. Neurosci.* **118**, 857–865 (2008). 18465429 10.1080/00207450701239459

[31] Klimesch, W. EEG alpha and theta oscillations reflect cognitive and memory performance: a review and analysis. *Brain Res. Brain Res. Rev.* **29**, 169–195 (1999). 10209231 10.1016/s0165-0173(98)00056-3

[32] Min, B. C., Chung, S. C., Min, Y. K. & Sakamoto, K. Psychophysiological evaluation of simulator sickness evoked by a graphic simulator. *Appl. Ergon.* **35**, 549–556 (2004). 15374762 10.1016/j.apergo.2004.06.002

[33] Hasselmo, M. E. What is the function of hippocampal theta rhythm?--Linking behavioral data to phasic properties of field potential and unit recording data. *Hippocampus* **15**, 936–949 (2005). 16158423 10.1002/hipo.20116

[34] Hasselmo, M. E., Hay, J., Ilyn, M. & Gorchetchnikov, A. Neuromodulation, theta rhythm and rat spatial navigation. *Neural Netw. Off J. Int. Neural Netw. Soc.* **15**, 689–707 (2002). 10.1016/s0893-6080(02)00057-6 12371520

[35] O’Keefe, J. & Burgess, N. Theta activity, virtual navigation and the human hippocampus. *Trends Cogn. Sci.* **3**, 403–406 (1999). 10529792 10.1016/s1364-6613(99)01396-0

[36] Watrous, A. J. et al. A comparative study of human and rat hippocampal low-frequency oscillations during spatial navigation. *Hippocampus* **23**, 656–661 (2013). 23520039 10.1002/hipo.22124 PMC4068262

[37] Lega, B. C., Jacobs, J. & Kahana, M. Human hippocampal theta oscillations and the formation of episodic memories. *Hippocampus* **22**, 748–761 (2012). 21538660 10.1002/hipo.20937

[38] Antonenko, P., Pasha, Paas, F., Grabner, R. & Gog, T. Using Electroencephalography to measure cognitive load. *Educ. Psychol. Rev.* **22**, 425–438 (2010).

[39] Friedman, N., Fekete, T., Gal, K. & Shriki, O. EEG-Based prediction of cognitive load in intelligence tests. *Front. Hum. Neurosci.* **13**, 191 (2019). 31244629 10.3389/fnhum.2019.00191 PMC6580143

[40] Kober, S. E. & Neuper, C. Sex differences in human EEG theta oscillations during spatial navigation in virtual reality. *Int. J. Psychophysiol. Off J. Int. Organ. Psychophysiol.* **79**, 347–355 (2011). 10.1016/j.ijpsycho.2010.12.002 21146566

