# Supplementary material for: Reduction in EEG theta power as a potential marker for spatial disorientation during flight
Source: Sci Rep. 2025 Jan 11;15:1682. doi: 10.1038/s41598-025-85219-4 (PMC11724846; doi:10.1038/s41598-025-85219-4)
Supplement: Supplementary file 1 — Supplementary Material 1 [file 41598_2025_85219_MOESM1_ESM.pdf]

Reduction in EEG Theta Power as a Potential Marker for Spatial Disorientation during Flight

Supplementary Information

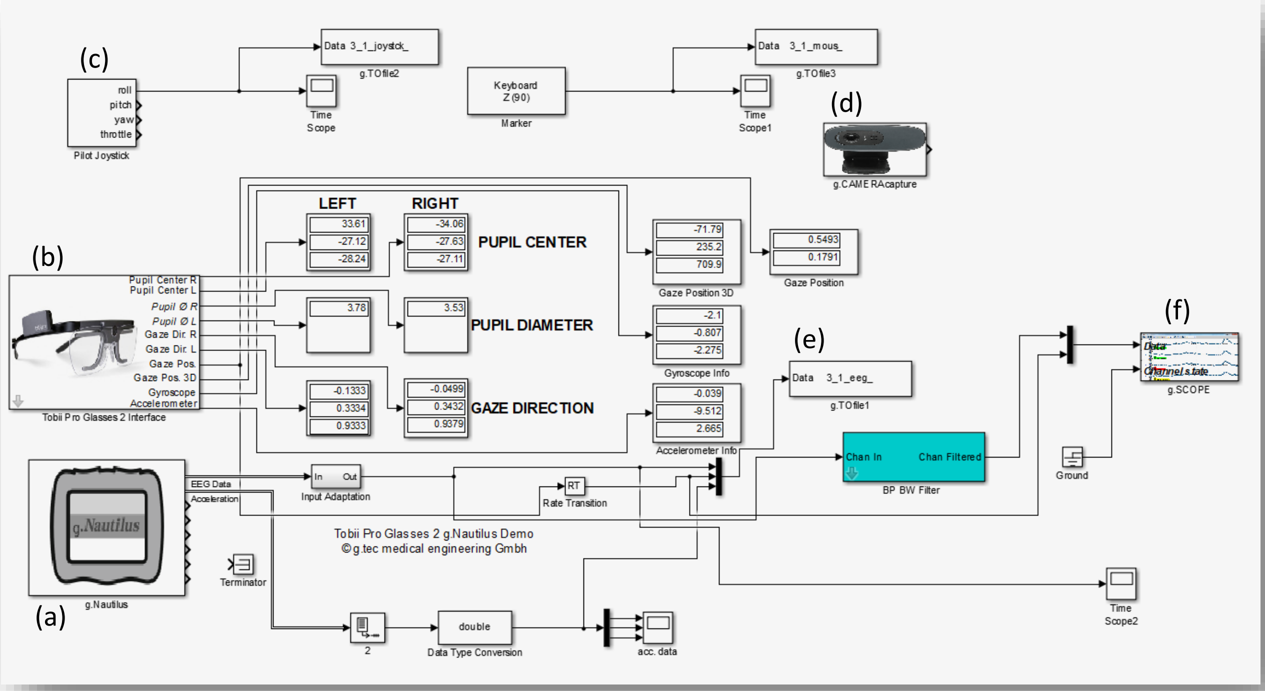

Figure 1: Simulink panel configuration used for data synchronization in the experiment.

This schematic layout illustrates the synchronization of data from all sensors onto the same timeline. The following data components were recorded on a 1/100-second timeframe chart:

(a) EEG recording system 'Nautilus wireless biosignal acquisition system,' a wearable system with 32 active dry electrodes (g.Tec, Austria). The g.Nautilus transmitted data via the 2.4 GHz band at a range of up to 10 m, with a sampling rate of 250 Hz.

(b) Eye Tracker data were collected using Tobii Pro Glasses 2. This wearable wireless system transmitted data via the 5 GHz band at a range of up to 20 m, with a sampling gaze rate of 100 Hz, recorded by 2 cameras per eye. Each subject underwent a calibration procedure before each test run. The system was adapted by the manufacturer to work with other wearable recording systems such as the g.Nautilus EEG set.

(c) Subjects reported their movement sensations using an ACGAM R1 remote mini joystick controller, which sampled signals at a rate of 250 Hz. The wireless pointing device transmitted data via Bluetooth 4.0 standard technology (~2.4 GHz band) at a range of up to 10 m. The device was sensitive to any apparent displacement from the center position. Subjects were instructed to mark their sensations by shifting the joystick pointer in the direction of the circular motion they felt and to return the joystick to the center position when the sensation extinguished.

(d) Chair position was documented by a Logitech C270 Widescreen HD remote camera at a rate of 30 frames per second (fps). The camera was sensitive to motion detection and documented the chair's rotation from a close range whenever motion was present.

(e) All data were collected into a single file and presented in real time on a wide screen.
